# Fluorescence of Atomic Germanium – Solution of Excitation Transfer

**DOI:** 10.1007/s10895-025-04220-2

**Published:** 2025-02-25

**Authors:** Pavel Dvořák, Vishal Dwivedi, Martina Mrkvičková, Nima Bolouki, Tomáš Medek, Milan Svoboda, Franklin Vaca Velásquez, Jan Kratzer, Jiří Dědina

**Affiliations:** 1 Department of Plasma Physics and Technology, Faculty of Science, Masaryk University, Kotlářská 2, Brno, 611 37 Czech Republic; 2 Department of Experimental Physics, Faculty of Mathematics, Physics and Informatics, Comenius University, Šafárikovo nám., Bratislava, 814 99 Slovak Republic; 3 Photonics Laboratory, Physics Unit, Tampere University, Korkeakoulunkatu 3, Tampere, 337 20 Finland; 4 Institute of Analytical Chemistry, Czech Academy of Sciences, Veveří, Brno, 602 00 Czech Republic

**Keywords:** Fluorescence, Germanium, Excitation transfer, Flame, Atomization

## Abstract

Laser induced fluorescence was used for detection of free germanium atoms in a miniature diffusion flame atomizer. Two excitation schemes were tested, which were based on excitation wavelengths around 250 nm and 205 nm. The second scheme suffered from a fast collisionally induced excitation transfer to another radiative state, which could led to an erroneous overestimation of Ge concentration by an order of magnitude. The excitation transfer was quantified and the problem was solved by means of measurement of temporally resolved fluorescence spectra. The equations for evaluation of fluorescence measurements affected by an excitation transfer to radiative states are presented. The obtained Ge concentration profiles revealed a strongly inhomogeneous distribution of free Ge atoms and a low germane atomization efficiency in the flame.

## Introduction

Fluorescence is, due to its sensitivity, versatility, and high temporal and spatial resolution, an important detection method in most scientific branches, as demonstrated e.g. by [1–8]. Usually, it is used only for detection of a presence of investigated species or for measurement of its relative concentration. To use fluorescence for measurement of absolute concentration directly in the environment probed by the excitation light, it is sometimes possible to calibrate the method by fluorescence signal generated in a standard with known concentration of investigated species [9]. Unfortunately, this calibration method is in its simple form restricted to detection of stable species and to the situations, when the fluorescence quantum yield (QY, i.e. the probability that absorption of an exiting photon leads to the emission of a fluorescence photon) in the investigated sample and in the standard do not differ. However, the intensity of the fluorescence signal can be used for direct measurement of absolute concentration even in situations when it is not possible to use a standard, e.g. in case of detection of unstable reactive species [7, 8]. The challenging part of such measurements is the determination of the fluorescence QY. Fortunately, when no excitation transfer to other radiative states occurs, QY can be determined from the lifetime of the directly excited state by the equation $QY = A\tau$, where *A* is the Einstein coefficient (the rate constant) of the spontaneous emission of the fluorescence photons from the directly excited state and $\tau$ is the lifetime of this state.

In liquid samples, the use of the above-mentioned equation for QY determination is strongly limited due to a number of channels that transfer the excitation to other radiative states, including rotational energy transfer, vibrational energy transfer, internal conversion or intersystem crossing [10]. In gases, the excitation transfer is much slower and the QY can be often obtained from the measurement of the lifetime [11, 12]. However, the excitation transfer frequently appears also in gaseous environments. Namely, the collisionally-induced rotational and vibrational energy transfers are known to complicate the fluorescence measurement of molecular species [13–15]. When free atoms are investigated, it is usually expected that it is possible to neglect any fluorescence signal originating from a state that would be populated by an excitation transfer. The present contribution demonstrates that this assumption is not always valid and presents the solution of such a complication.

The present work deals with laser-induced fluorescence of free germanium atoms. Germanium is one of Technology Critical Elements - rare in nature but vital for modern technologies. Therefore, it is necessary to develop analytical methods for its determination at very low concentrations [16] to be able to assess the influence of anthropological activities on Ge levels in the environment. Although fluorescence detection of atomic Ge was realized by several works [17–21], fluorescence measurement of its absolute concentration directly in the volume probed by the excitation radiation was realized in none of them. The works [17] and [18] used the laser-induced excitation of Ge atoms to the states 4 s$^2$4p5s $^1$P$^{\circ }$ $J=1$ and 4 s$^2$4p5s $^3$P$^{\circ }$ $J=1$ by photons with wavelengths 253 nm and 269 nm, respectively. These works were not focused to the determination of absolute concentration of free Ge atoms in the used flame or graphite furnace and it would be difficult to use their experiments for such a purpose, because both these works excited Ge atoms from the $J=1$ level, so they did not probe the ground level of atomic germanium. In order to enable direct fluorescence measurement of absolute concentration of free Ge atoms, the present work introduces and compares two Ge excitation schemes.

Free germanium atoms were produced by atomization of germanium hydride (germane, GeH$_4$). Conversion of germanium ions present in liquid sample into a volatile species, i.e. germanium hydride, is advantageous approach since it offers almost 100 % analyte introduction efficiency into the detector while it enables analyte separation from the liquid matrix at the same time minimizing the risk of interferences. In the following detection step of element analysis, it is essential to dissociate the hydride molecule in order to form ground state Ge atoms, that can be directly detected by atomic absorption spectroscopy (AAS) or atomic fluorescence spectroscopy (AFS). Atomization of hydrides can be reached in miniature flames, ambient plasmas or in heated quartz tubes [22, 23]. It was shown recently that in all types of hydride atomizers the atomization proceeds via hydrogen radical mechanism [23–25]. In case of GeH$_4$ atomization, the main reactions are H + O$_2$ $\leftrightarrow$ OH + O, O + H$_2$ $\leftrightarrow$ OH + H, OH + H$_2$ $\leftrightarrow$ H$_2$O + H, GeH$_n$ + H $\leftrightarrow$ GeH$_{n-1}$ + H$_2$. The most common hydride atomizer in AFS, applicable also in AAS, is the diffusion flame (DF), which is based on a miniature diffusion Ar – H$_2$ flame that generates hydrogen radicals and atomizes the analyte hydride.

In contrast to other hydride forming elements (As, Se, Te, Pb, Bi, Sn, Sb), the sensitivity obtained for Ge using AAS with the DF is significantly, at least by an order of magnitude, lower [26, 27]. Nevertheless, germane introduction efficiency into the atomizer/detector, i.e., the yield of the hydride generation (HG) step, was found satisfactory in case of Ge, reaching at least 80 % according to [26] and even almost 100 % according to our yet unpublished data. As a consequence, low atomization efficiency of germane in the DF atomizer is suspected to be the reason for low sensitivity of Ge determination by HG-AAS. Therefore, a more detailed insights into the atomization processes of GeH$_4$ in the DF, including quantification of germane atomization efficiency, spatial distribution of free Ge atoms in the flame and their fate is clearly needed to understand the reason for low sensitivity of DF atomizer coupled to AAS or AFS detector. From this reason, the presented work was realized in the diffusion flame atomizer.

## Method

The method used in this paper utilises measurement of the concentration of investigated species directly from the intensity of the fluorescence signal without necessity to use a standard with known concentration [7, 8]. The species are excited (usually from their ground state, but detection of other states is also possible) by photons (usually by a laser beam), the following fluorescence is detected (usually by an ICCD camera or a photomultiplier) and its intensity is used for the determination of the concentration of the investigated species. The detector is usually covered by an interference filter, which transmits the fluorescence radiation but blocks the scattered (laser) photons and most of eventual background radiation. The following text describes the situation, when a pulsed laser excitation is used.

The method is based on the following relation between the intensity of the fluorescence signal ($M_f$) and the concentration of detected species in the probed state (*n*):

1 $$\begin{aligned} M_f \,\,=\,\, n\,\frac{\kappa B_{G \rightarrow E}}{c}\, E_f \, A_E \, \tau \, C_f, \end{aligned}$$

where $B_{G \rightarrow E}$ is the Einstein coefficient for the excitation from the ground state to the excited state, *c* is the speed of light, $E_f$ is the mean energy of laser pulses, $A_E$ is the Einstein coefficient for the spontaneous emission of the measured fluorescence photons by transition from the excited state to a lower state and $\tau$ (already introduced in Sect. “Introduction”) is the fluorescence lifetime. $\kappa$ describes the spectral overlap between the laser line and the absorption line – for a laser with narrow spectral line it is equal to the ratio between the maximum and the integral intensities of the absorption line [28]. $M_f$ in the Eq. 1 is the fluorescence signal integrated temporally over the whole fluorescence duration and spectrally both over the whole fluorescence transition and over the whole excitation line. The quantity $C_f$ describes the sensitivity of the particular experimental setup. It depends on the sampled volume, solid angle for detection of fluorescence photons covered by the detector, transmission of the used interference filter, transmission of all the optical elements placed in the detection path, quantum efficiency and complete sensitivity of the detector, eventually on the number of accumulations of the collected signal [29].

It is often difficult to predict the absolute value of the sensitivity $C_f$. Therefore, its value is usually calibrated. Fortunately, it is not necessary to perform the calibration on a standard with investigated species with known concentration, because such a method would be complicated in case of unstable species. Frequently, a simple Rayleigh scattering [30] on a gas with known pressure, e.g. on Ar or on the ambient air is used. The intensity of the measured Rayleigh signal is given by

2 $$\begin{aligned} M_r \,\,=\,\, n_r \, 4 \pi \,\frac{{\text{ d }} \sigma _r}{{\text{ d }} \Omega }\, \, \frac{E_r}{h\nu _r} \, C_r, \end{aligned}$$

where $n_r$ is the concentration of the gas used for calibration, ${\text{ d }} \sigma _r / {\text{ d }} \Omega$ is the differential cross section for Rayleigh scattering on the gas, $E_r$ is the mean energy of laser pulses used for Rayleigh scattering, $\nu _r$ is the frequency of laser light, *h* is the Planck constant. $C_r$ is the detection sensitivity for the Rayleigh wavelength. The ratio of the detection sensitivities for fluorescence and Rayleigh photons can be obtained from usually known constants by $C_f / C_r = t_f\, \eta _f / \eta _r$, where $t_f$ is the transmission of the interference filter used for the detection of fluorescence (which is removed for the measurement of Rayleigh scattering), $\eta _f$ and $\eta _r$ are quantum efficiencies of the detector for the fluorescence and Rayleigh wavelengths, respectively.

The combination of the Eqs. 1 and 2 leads to the following equation for the concentration of studied species:

3 $$\begin{aligned} n \,\,=\,\, \frac{M_f}{M_r} \,\frac{C_r}{C_f}\, \frac{p_r}{k T_r} \, \frac{E_r}{E_f}\, \frac{1}{h \nu _r} \, 4\pi \,\frac{{\text{ d }} \sigma _r}{{\text{ d }} \Omega } \, \frac{c}{A_E \,\tau \, \kappa B_{G \rightarrow E}}, \end{aligned}$$

where $p_r$ and $T_r$ are the pressure and temperature of the calibration gas, respectively, *k* is the Boltzmann constant (i.e. $n_r=p_r/kT_r$).

When atomic species are investigated, the situation is relatively simple, because their ground state comprises only one or few levels. By contrast, when molecular species are detected, the full rotational (and eventually also vibrational) structure of the ground state must be taken into account [31].

**Fig. 1:**
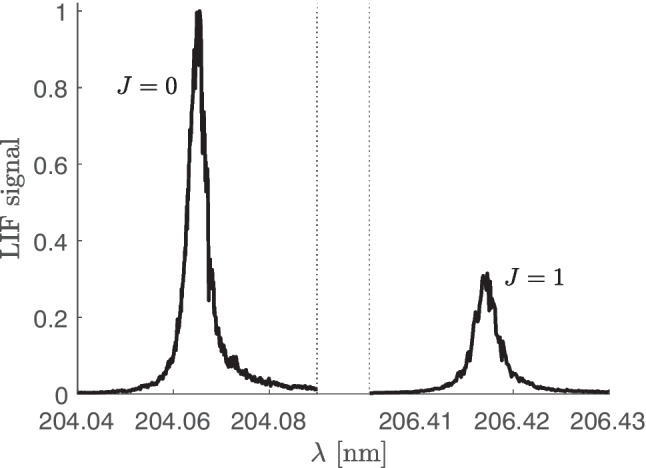
An example of the excitation spectrum of Ge atoms with two lines - excitation from two levels, $J=0$ and $J=1$, of the ground state 4 s$^2$4p$^2$ $^3\!$P. (The laser wavelength shown on the horizontal axis deviates a little from the reality due to a systematic shift of the reading of the position of the grating in the used dye laser)

The fluorescence signal $M_f$ in the Eq. 1 is spectrally integrated over the whole excitation line. In our case, i.e. for measurement in a gas phase with a narrow-line laser, it is usually easy to distinguish a fine (or eventual rotational) structure of the transition. Two germanium lines of our excitation spectrum are shown in the Fig. 1. The distinguishing of the two lines enabled to obtain separate integrals of these lines and to get the concentration of each of the levels $J=0$ and $J=1$, as described bellow in the Section “Results”. If the excitation lines were overlapped due to a strong broadening (as happens e.g. in liquids) or due to a use of a broad-band excitation source, it would be necessary to take into account that several lines would contribute to the resulting peak.

**Fig. 2:**
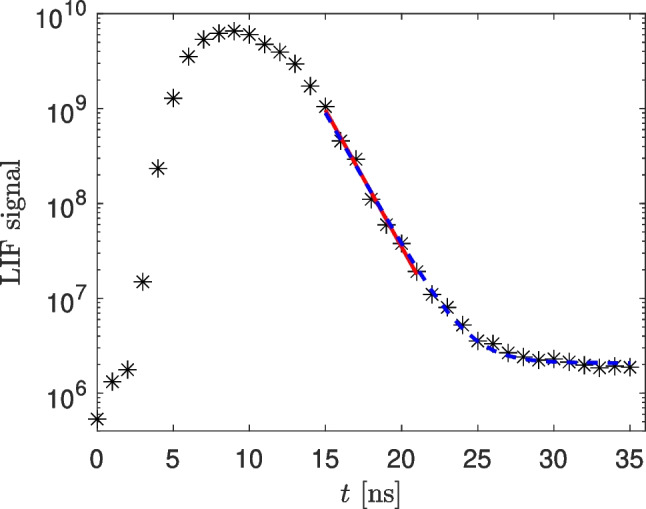
An example of the measurement of the lifetime of excited Ge atoms. The exponential decay was fitted after the end of the laser pulse (i.e. from $t=15$ ns). The red solid line shows the simple exponential fit in the range which is not influenced by the background. The broken blue line shows the fit that takes into account the background signal

In order to use the Eq. 3, it is necessary to determine the lifetime of the excited state (i.e. the fluorescence decay time), as it is directly connected to the fluorescence QY. An example of our measurement of the fluorescence decay of Ge atoms excited to the 4 s$^2$4p4d $^3$D $J=1$ state is shown in the Fig. 2. The depicted measurement was realized by an ICCD camera synchronized with the laser. The gate delay of the camera was varied in order to get the fluorescence signal in various phases of the fluorescence process. In this case, the lifetime can be obtained from a simple exponential fit of the measured decay after the end of the laser pulse, as shown by the red line. Eventually, if there is some background signal induced by the laser pulse that can not be subtracted by measurement of a dark image, an exponential decay with an added constant background signal can be fitted to the measured data, as shown by the blue curve. In the example shown in the Fig. 2 both these methods gave the same lifetime value 1.6 ns. In situations, when the temporal tail of the laser pulse disturbs practically the whole fluorescence decay, it would be necessary to fit a convolution of the temporal profile of the laser pulse with an exponential decay, as described in [29]. (It should be noted, that the measurement shown in the Fig. 2 can not be directly used for the determination of the lifetime of the directly excited 4 s$^2$4p4d $^3$D $J=1$ state, as this measurement was affected by an excitation transfer to another radiative state, as described below in the Section “Results”.)

The measurement may suffer saturation, i.e. a decrease of the intensity of the fluorescence signal from its linear dependence on the energy of laser pulses, which may emerge at high laser power. The processes guilty for saturation are the depletion of the ground state by laser excitation, stimulated emission from the excited state and eventually also the photoionization of the excited state. Various points in the laser beam may suffer different saturation due to the nonhomogeneity of the laser beam. Evaluation of fluorescence measurement realized in the partially saturated regime was introduced in [32].

Finally, it should be noted that when a substantial part of the excitation beam is absorbed (or scattered) in the sample, the fluorescence intensity from the front and back parts of the sample may differ due to different intensity of the laser beam. Not only fluorescence, but also the absorption process can be partially saturated. Such a situation is solved in [29].

## Experimental

The presented approach will be demonstrated by the case study of the efficiency of the diffusion flame atomizer for the dissociation of GeH$_4$.

### Diffusion flame atomizer

Germane was produced in an in-house made, three-channel hydride generation system based on a peristaltic pump (Ismatec, Switzerland), identical to that described in [26]. A three-way valve was used to switch between blank and Ge standard solution (2 mg/l Ge) introduction in the sample channel. Standard or blank flow (1.0 ml/min) was merged with a stream of the reductant (NaBH$_4$) solution delivered at a flow rate of 1.0 ml/min. The reaction mixture was merged downstream with a flow of carrier Ar (75 ml/min) controlled by a mass flow controller (Omega Engineering, USA) and directed to the quartz gas-liquid separator (GLS) with a forced outlet. The liquid waste was drained by the same peristaltic pump from its bottom. Apart from the germane generated, the gas phase directed from the GLS to the diffusion flame atomizer by a stream of Ar carrier gas contains 8 ml/min H$_2$ produced as a side-product of NaBH$_4$ decomposition as found experimentally and some amount of co-generated aerosol/water vapor.

**Fig. 3:**
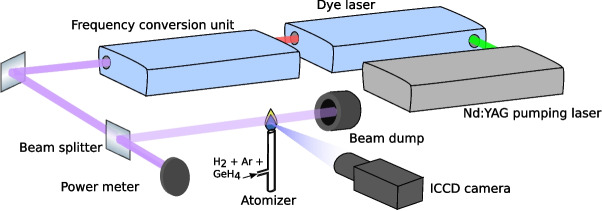
Experimental setup for the first set of measurements. For the other setup, the dye laser system was replaced by an OPO system. For measurement of fluorescence spectra, the ICCD camera was replaced by an optical fibre providing the fluorescence signal to the spectrometer

**Fig. 4:**
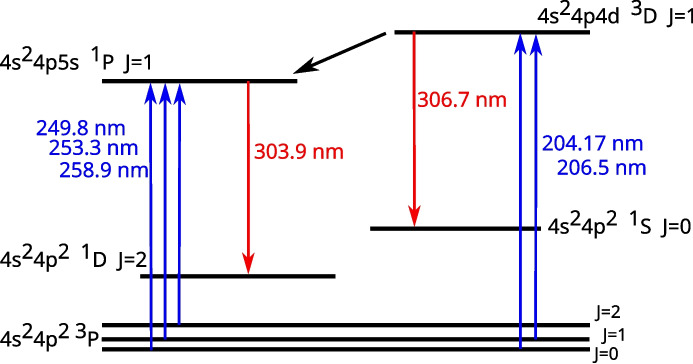
Excitation scheme of the fluorescence of Ge atoms

The diffusion flame (DF) atomizer [22] consisted of a vertical 150 mm long quartz support tube (6.0 mm i.d., 8.0 mm o.d.). Its bottom part was sealed with the gas phase being supplied through a 35 mm long side inlet arm (2.0 mm i.d., 4.0 mm o.d.) made of quartz, fused to the vertical support tube 50 mm from its bottom. Two polypropylene T-connectors (union tees 1/8”, Cole-Parmer, USA) were employed upstream the inlet arm of the DF atomizer to mix additional argon flow rate (625 ml/min Ar) with hydrogen as a fuel for the diffusion flame (292 ml/min H$_2$) in the first T-connector and subsequently to add the gas phase from the GLS of the hydride generator containing analyte hydride together with 75 ml/min Ar + 8 ml/min H$_2$ co-generated. As a consequence, the total gas flow rate delivered to the diffusion flame burning at the top of the support tube was 1 000 ml/min with a composition of 70 % Ar and 30 % H$_2$.

All reagents were of analytical grade or higher purity. Tris buffer (termed TRIS–HCl further in the text) was prepared from Trizma hydrochloride (Sigma Aldrich, Germany) with pH adjusted by NaOH (Sigma Aldrich, Germany) to 6.5. Ethylendiamintetraacetic acid disodium salt dihydrate (EDTA) produced by Lachema, Czech Republic was added into the 0.25 mol/l TRIS–HCl buffer to reach its 0.005 mol/l concentration. This solution served as a blank and a matrix to prepare Ge standard solution. The reductant was 3 % solution of NaBH$_4$ (Sigma Aldrich, Germany) in 0.1 % (m/v) KOH (Lach-Ner, Czech Republic). Working standard of 2 mg/l Ge was prepared fresh daily from its stock solution (1 000 mg/l Ge(IV), Astasol Analytika, Czech Republic) by dilution with blank. The gases used Ar (99.996 %) and H$_2$ (99.999 %) were purchased from Messer Technogas s.r.o.

### Laser-Induced Fluorescence

The scheme of the laser-induced fluorescence setup is shown in Fig. 3. Two different laser systems were used in the two sets of experiments. First, a tunable dye laser (Sirah, PrecisionScan PRSC-D-24-EG) pumped by a pulsed Q-switched Nd:YAG laser (Quanta-Ray PRO-270-30) produced laser pulses with a spectral width of 0.4 pm, duration of 8 ns, and repetition rate of 30 Hz. Second, an OPO laser (EKSPLA PG411-SH/DUV) pumped by a Nd:YAG laser (EKSPLA PL2231-50-TRAIN) generated pulses with a spectral width of 20 pm, duration of 15 ps, and repetition rate of 50 Hz. We used various laser wavelengths in range 204 – 260 nm to excite the germanium atoms to two various states, 4 s$^2$4p4d $^3$D $J=1$ and 4 s$^2$4p5s $^1$P $J=1$, as shown in Fig. 4 and discussed bellow in the Sect. “Results”. The laser beam passed through a silica plate acting as a beam splitter – a large fraction was transmitted to the pyroelectric power meter (Ophir, Vega PE9), while only 10 % of the laser energy (in order to reduce the saturation of the fluorescence process) was reflected and continued to the atomizer. The beam passed over the centre of the flame and ended up in the beam dump.

We observed the fluorescence generated by the laser beam at the height of 2 mm above the top of the support tube. The fluorescence radiation was detected by an ICCD camera (Princeton Instruments, PI-MAX 1024) perpendicular to the laser beam. An interference filter (AHF F39-320 320/40 BrightLine HC) was mounted on the camera lens to separate the fluorescence signal from the scattered laser radiation or flame emission. In the case of spectrally resolved detection, the fluorescence radiation was focused by a lens onto the optical fibre and directed to the Jobin-Yvon FHR 1000 spectrometer equipped with another ICCD camera.

## Results

In order to find the concentration of free Ge atoms and to determine the atomization efficiency of GeH$_4$ in the flame, we excited Ge atoms from their ground state by laser photons with wavelength 204.17 nm to the 4 s$^2$4p4d $^3$D $J=1$ state. However, the Ge ground state is a triplet state with energy levels 0 eV (the ground $J=0$ level), $E_{J=1}=0.069$ eV (the $J=1$ level) and $E_{J=2}=0.175$ eV (the $J=2$ level) [33]. The temperature in the flame is expected to be sufficiently high to excite also the levels $J=1$ and $J=2$, so it is not sufficient to measure only Ge atoms in the ground level ($J=0$), because a significant part of the atoms may be hidden in the remaining two levels. In order to determine what is the fraction of Ge atoms in the ground level (the Boltzmann fraction), we excited Ge atoms also from the $J=1$ level by laser photons with wavelength 206.5 nm. The ratio between the fluorescence signal obtained with excitation from the levels $J=1$ and $J=0$ can be used to the determination of the excitation temperature of Ge atoms according to the equation

4 $$\begin{aligned} \frac{M_{f, J=1}}{M_{f, J=0}} \,\,=\,\, \frac{B_{J=1 \rightarrow E}}{B_{J=0 \rightarrow E}}\, \frac{g_{J=1}}{g_{J=0}}\,\,\exp \!{\left( -\frac{E_{J=1}}{kT_{\textrm{exc}}} \right) }, \end{aligned}$$

where $g_{J=1}/g_{J=0} = 3$ is the ratio of the degenerations of the levels $J=1$ and $J=0$. The experimentally determined ratio $M_{f, J=1}/M_{f, J=0}$ corresponds, according to the Eq. 4, to the germanium excitation temperature $T_{\textrm{exc}} = 780$ K. It should be mentioned that this is a value averaged along the exciting laser beam over the flame region rich on free Ge atoms, since the temperature distribution in the actual flame is not homogeneous [34]. The obtained temperature can be used for the calculation of the Boltzmann fraction

5 $$\begin{aligned} f_B \,=\, \frac{1}{1\,+\, 3 \textrm{e}^{\left( - \frac{E_{J=1}}{kT_{\textrm{exc}}} \right) } \,+\, 5 \textrm{e}^{\left( - \frac{E_{J=2}}{kT_{\textrm{exc}}} \right) }}, \end{aligned}$$

where 1, 3 and 5 are the degenerations of the levels $J=0$, 1 and 2, respectively. The corresponding Boltzmann fraction of free atoms in the ground level $J=0$ was $f_B = 0.41$.

**Fig. 5:**
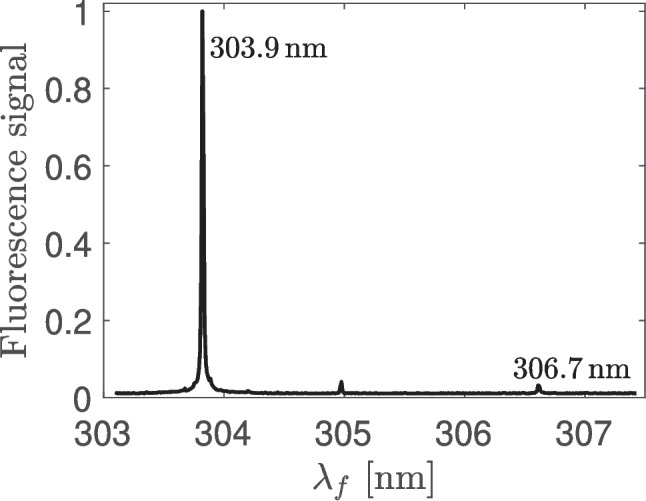
Fluorescence spectrum measured when the Ge state 4 s$^2$4p4d $^3$D, $J=1$ was directly excited

Before we used the intensity of the fluorescence signal for calculation of the concentration of free Ge atoms, we measured the fluorescence spectrum. In the region used for the detection (290 – 310 nm) we expected to see (only) the fluorescence line with the wavelength 306.7 nm. However, as shown in the Fig. 5, this expected line was very weak and most of the measured fluorescence signal was formed by another line with the wavelength 303.9 nm. Photons of this line originate from the excited singlet state 4 s$^2$4p5s $^1$P $J=1$, which was not directly excited by the laser. It means that the measurement suffered a fast collisional transfer of the excitation and it was not possible to use the Eq. 3 for the calculation of Ge concentration.

In order to get quantitative results from the measurement disturbed by the excitation transfer, we wrote the rate equations for such a fluorescence process. When all the saturation processes are neglected, the evolution of the concentration of the directly excited state ($n_E$, in our case the 4 s$^2$4p4d $^3$D $J=1$ state) and the concentration of the state populated by the excitation transfer ($n_T$, in our case the 4 s$^2$4p5s $^1$P $J=1$ state) can be described by

6 $$\begin{aligned} \frac{{\text{ d }} n_E}{{\text{ d }} t}= &   n\,\frac{\kappa B_{G \rightarrow E}}{c}\,I(t) \,-\, \frac{n_E(t)}{\tau _E} \end{aligned}$$

7 $$\begin{aligned} \frac{{\text{ d }} n_T}{{\text{ d }} t}= &   T \,n_E(t) \,-\, \frac{n_T(t)}{\tau _T}. \end{aligned}$$

In these equations, *I* is the laser intensity (irradiance), *T* is the rate of the excitation transfer from the state 4 s$^2$4p4d $^3$D $J=1$ to the state 4 s$^2$4p5s $^1$P $J=1$, $\tau _E$ and $\tau _T$ are the lifetimes of these two states.

The total amount of fluorescence photons emitted from a unit volume due to one laser pulse can be calculated by temporal integration of the populations of the excited states. Such amounts of photons ($F_E$ and $F_T$) emitted on the fluorescence spectral lines 306.7 nm and 303.9 nm, respectively, can be expressed from Eqs. 6 and 7, respectively:

8 $$\begin{aligned} F_E= &   \int \limits _0^{\infty } A_E\,n_E\, {\text{ d }} t \,=\, A_E\,\tau _E \, \frac{\kappa B_{G \rightarrow E}}{c} \, \int \limits _0^{\infty } n\, I(t) {\text{ d }} t \end{aligned}$$

9 $$\begin{aligned} F_T= &   \int \limits _0^{\infty } A_T\,n_T\, {\text{ d }} t \,=\, A_T\,\tau _T \,T \, \tau _E \, \frac{\kappa B_{G \rightarrow E}}{c} \, \int \limits _0^{\infty } n\, I(t) {\text{ d }} t. \end{aligned}$$

Here $A_E$ and $A_T$ are the Einstein coefficients for spontaneous emission for the lines 306.7 nm and 303.9 nm, respectively. When these two equations are multiplied by the detection sensitivities ($C_E$ and $C_T$) for the two lines, spatially integrated over the sampled volume and summed together, we get the relation for the fluorescence signal acquired from both the lines:

10 $$\begin{aligned} M_f \,\,=\,\, n\,\frac{\kappa B_{G \rightarrow E}}{c}\,E_f \, \tau _E \,\left( C_E\, A_E \,+\, C_T\,A_T\,\tau _T\,T \right) . \end{aligned}$$

Now, when the Rayleigh calibration is applied, we get the final equation for the concentration of measured species

11 $$\begin{aligned} n \,\,=\,\, \frac{M_f}{M_r} \,\frac{C_r}{C_E\,A_E\,+\,C_T\,A_T\,\tau _T\,T}\, \frac{1}{\tau _E} \,\frac{p_r}{k T_r} \, \frac{E_r}{E_f}\, \frac{1}{h \nu _r} \, 4\pi \,\frac{{\text{ d }} \sigma _r}{{\text{ d }} \Omega } \, \frac{c}{\kappa B_{G \rightarrow E}}, \end{aligned}$$

that, in contrast to the Eq. 3, takes into account the excitation transfer to another radiative state which also contributes to the measured fluorescence signal. For our particular transitions and experimental conditions we used the values $A_E = 4.5 \cdot 10^6$ s$^{-1}$ [35], $A_T = 2.8 \cdot 10^8$ s$^{-1}$ [33], $B_{G \rightarrow E} = 1.69 \cdot 10^{20}$ m$^3$/Js$^2$ (derived from $A_{E \rightarrow G} = 1.1 \cdot 10^8$ s$^{-1}$ [33]), $\kappa = 2.9 \cdot 10^{-11}$ s and for wavelength 204.17 nm (i.e. frequency $1.47 \cdot 10^{15}$ Hz) we used ${\text{ d }} \sigma _r/{\text{ d }} \Omega = 3.76 \cdot 10^{30}$ m$^2$ [30].

**Fig. 6:**
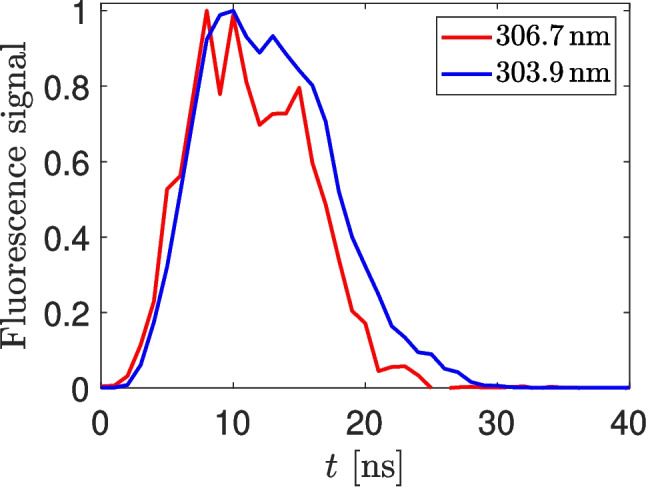
The temporal evolution of the intensity of the fluorescence lines 303.9 nm and 306.7 nm measured when the Ge state 4 s$^2$4p4d $^3$D, $J=1$ was directly excited. The data were normalized to a maximum of 1. Before normalization, the intensity of the 306.7 nm line was 35 $\times$ lower than that of the 303.9 nm line

When the Eq. 11 should be applied, it is necessary to know not only the lifetime of the directly excited state ($\tau _E$), but also the two new constants *T* and $\tau _T$. In order to determine their values, we realized temporally resolved measurement of fluorescence spectra. The obtained temporal evolution of the intensities of both the fluorescence lines is shown in the Fig. 6. The weak emission of the directly excited 4 s$^2$4p4d state (306.7 nm, shown by the red noisy curve) a little precedes the emission of the 4 s$^2$4p5s state (303.9 nm, the blue line). In order to get the lifetime of the directly excited state, we fitted the exponential decay (i.e. the solution of the Eq. 6 for $I=0$) to the tail of the red curve after the end of the laser pulse and we obtained the value $\tau _E = 2$ ns. In order to obtain the remaining two constants, we fitted the solution of the Eq. 7 to the blue curve. The concentrations $n_E$ and $n_T$ were expected to be directly proportional to the intensities of the lines 306.7 nm and 303.9 nm, respectively. We obtained values $T = 3.6 \cdot 10^8$ s$^{-1}$ and $\tau _T = 1.6$ ns. It means that the quantum yield of the excitation transfer ($T\cdot \tau _E$) in our experiment was approximately 70 %. If this effect was ignored and the Eq. 3 with fluorescence signal $M_f$ spectrally integrated over both radiative transitions was used instead of the Eq. 11, it would lead to the overestimation of germanium concentration by a factor $\sim 20$. (It should be noted that the quantities $\tau _E$, $\tau _T$ and *T* depend on the gas composition, pressure and temperature, i.e. the listed values are valid only in our experimental conditions.)

**Fig. 7:**
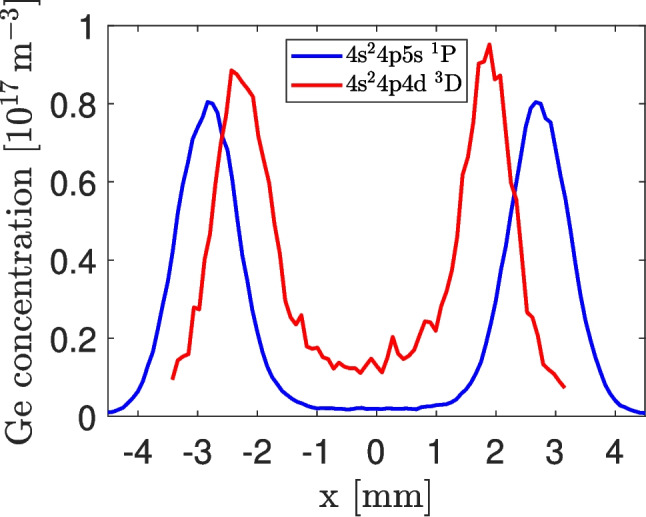
Concentration of atomic Ge measured in an Ar – H$_2$ – GeH$_4$ flame in a horizontal line situated 2 mm above the edge of the burner. The red and blue curves were obtained from measurements with laser-induced excitation of Ge atoms to the states 4 s$^2$4p4d $^3$D and 4 s$^2$4p5s $^1$P, respectively

Finally, the resulting concentration profile of the germanium atoms measured 2 mm above the burner is shown in the Fig. 7 by the red curve. The maximum concentration was found at the flame edges, i.e. in the hot combustion zone where hydrogen reacts with oxygen coming from the ambient air. The concentration of the free Ge atoms in the relatively cold [34] flame center is significantly smaller. However, it should be noted that the concentration values in the flame center obtained in this particular measurement suffer a high uncertainty, because the values of the constants $\tau _E$, $\tau _T$ and *T* were acquired from the stronger signal originating from the flame edges. Their values in the hot combustion zone and cold flame center with different gas composition may differ, which may lead to deflected concentration values in the flame center.

The results above were obtained with the nanosecond dye laser. Later, we acquired the picosecond OPO, which gave us an easy opportunity to excite the 4 s$^2$4p5s $^1$P $J=1$ state directly by laser photons with wavelength $\sim 250$ nm, as shown in the left part of the Fig. 4. Although these $^3$P $\rightarrow$ $^1$P transitions change the multiplicity, they are sufficiently strong to be used for excitation, as demonstrated by their values of the Einstein coefficient for spontaneous emission that reach the values $5.1\cdot 10^6$ s$^{-1}$ – $1.3\cdot 10^7$ s$^{-1}$ [33]. This excitation scheme did not suffer any fluorescence signal originating from any state populated by a collisional energy transfer, so it was possible to evaluate the measurements realized with this excitation scheme by the standard Eq. 3 and verify the results obtained in the previous experiment by means of the Eq. 11. The concentration values obtained with the direct excitation to the 4 s$^2$4p5s state in a similar burner are shown in the Fig. 7 by the blue curve. The values of the maximum concentration found in the combustion zone at the flame edge differ by approximately 10 %, which is a reasonable agreement demonstrating the validity of the approach described by the Eqs. 6 – 11. The disagreement in the width of the saddle structure of the curves shown in the Fig. 7 was caused by a slightly different supporting atomizer tube and a little different position of the laser beam when the experiment was later repeated with the new OPO laser system.

Distributions of free Ge atoms shown in Fig. 7, namely the blue curve obtained from the excitation by wavelengths around 250 nm, can be employed to estimate atomization efficiency of germane in the diffusion flame. At least at the height of 2 mm above the top of the support tube, the free atom distribution is strikingly inhomogeneous suggesting negligible atomization efficiency close to the vertical axis of the support tube of the atomizer. In contrast, maximum free atom concentration appeared in the combustion zone close to the flame edge. Comparing the maximum concentration values, around $0.8 \cdot 10^{17}$ atom/m$^3$, with expectations based on the employed experimental parameters (gas flow rates, analyte supply rate to the atomizer and flame temperature 780 K, and on the quantified hydride generation efficiency of 100 %, the atomization efficiency in the combustion zone close to the flame edge is as low as around 1.3 %.

## Conclusion

Two excitation schemes were tested for fluorescence detection of free Ge atoms: One is based on excitation of the 4 s$^2$4p5s $^1$P $J=1$ by wavelengths around 250 nm, which is followed by a strong fluorescence transition to the state 4 s$^2$4p$^2$ $^1$D $J=2$. The fluorescence is localised at 303.9 nm.

The other excitation scheme is based on the excitation of the 4 s$^2$4p4d $^3$D $J=1$ state by wavelengths around 205 nm. In our conditions (Ar – H$_2$ flame at atmospheric pressure) the 306.7 nm fluorescence of the directly excited state was weak, but there was a fast collisionally induced excitation transfer to the 4 s$^2$4p5s $^1$P $J=1$ state followed by a strong 303.9 nm fluorescence emission.

The rate equations were used to describe the fluorescence process that requires to take into account the radiation induced by the excitation transfer. It was shown that rate constants required for quantitative evaluation of such fluorescence measurement can be acquired from temporally resolved fluorescence spectra. Then, the Eq. 11 can be used for determination of the concentration. The absolute concentrations of germanium atoms measured by the two different excitation schemes were in a reasonable agreement. From reasons discussed above, we can recommend the scheme based on excitation around 250 nm as the more reliable.

The measured distribution of Ge free atoms was strongly inhomogeneous with maximum in the combustion zone at the flame edge. The measured values of Ge concentration were significantly lower than the concentration of the supplied GeH$_4$. The low atomization efficiency of GeH$_4$ observed in diffusion flame atomizer by LIF in this work is in agreement with low sensitivity of Ge determination by hydride generation atomic absorption spectrometry (HG-AAS) using DF as hydride atomizer. Moreover, the results presented in this work confirm the previously formulated hypothesis [26] expecting that atomization efficiency of germane in the DF is, in contrast to other hydride forming elements, extremely low. The data achieved in this work can be further applied in the field of analytical chemistry to improve existing hydride atomizers or find alternative ones.

## Data Availability

The data will be deposited in the system of Masaryk University.

## References

[1] Geddes CD, Lakowicz JR (2017) Reviews in Fluorescence 2016. Springer, Cham, Switzerland

[2] Birch DJS, Chen Y, Rolinski OJ (2015) fluorescence. In: Andrews DA (ed) Photonics: Biomedical Photonics, Spectroscopy, and Microscopy. John Wiley & Sons, New Jersey, pp 1–58

[3] Šachl R, Amaro M (2023) Fluorescense Spectroscopy and Microscopy in Biology. Springer, Cham, Switzerland

[4] Shashkova S, Leak MC (2017) Single-molecule fluorescence microscopy review: shedding new light on old problems. Biosci Rep 37:20170031 10.1042/BSR20170031 PMC5520217 28694303

[5] Jiang X, Yang R, Lei X, Xue S, Wang Z, Zhang J, Yan L, Xu Z, Chen Z, Zou P, Wang G (2024) J Fluoresc 34:965 37498366 10.1007/s10895-023-03344-7

[6] Abraham JE, Balachandran M (2022) J Fluoresc 32:887 35303239 10.1007/s10895-022-02915-4

[7] Kohse-Höinghaus K (1994) Prog. Energy Comb. Sci. 20:203

[8] Amorim J, Baravian G, Jolly J (2000) J Phys D Appl Phys 33:51

[9] Manaigo F, Chatterjee A, Bogaerts A, Snyders R (2024) Insight in no synthesis in a gliding arc plasma via gas temperature and density mapping by laser-induced fluorescence. Plasma Sources Sci Technol 33:075005

[10] Klán P, Wirz J (2009) Photochemistry of Organic Compounds. Wiley, Chichester

[11] Bittner J, Kohse-Höinghaus K, Meier U, Just T (1988) Chem Phys Lett 143:571

[12] Voráč J, Obrusník A, Procházka V, Dvořák P, Talába M Spatially resolved measurement of hydroxyl radical (OH) concentration in an argon RF plasma jet by planar laser-induced fluorescence. Plasma Sources Sci Technol 25011–12. 10.1088/0963-0252/23/2/025011

[13] Procházka V, Tučeková Z, Dvořák P, Kováčik D, Slavíček P, Zahoranová A, Voráč J (2018) Plasma Sourc Sci Technol 27:015001

[14] Copeland RA, Crosley DR (1984) Rotational level dependence of electronic quenching of OH (a). Chem Phys Lett 107:295

[15] Li L, Nikiforov A, Xiong Q, Britun N, Snyders R, Lu X, Leys C (2013) OH radicals distribution in an ar-ho atmospheric plasma jet. Phys Plasmas 20:093502

[16] Filella M, Rodríguez-Murillo JC (2017) Chemosphere 182:605 28525874 10.1016/j.chemosphere.2017.05.024

[17] Ezer M, Gondi R, Kennehan E, Simeonsson JB (2023) Determination of germanium species by hydride generation atomic absorption spectrometry: Comparison of atomizers based on diffusion flame, heated quartz tube, and dielectric barrier discharge plasma. At Spectrosc 44:207

[18] Aucélio RQ, Rubin VN, Becerra E, Smith BW, Winefordner JD (1997) Anal Chim Acta 350:231

[19] Dagnall RM, Kirkbright GF, West TS, Wood R (1970) Analyst 95:425

[20] Guo X, Guo X (1998) Analytica Chimica 373:303

[21] Jiang SL, Shi CH, Wu JG (2007) J Food Qual 30:481

[22] Dědina J (2007) Atomization of volatile compounds for atomic absorption and atomic fluorescence spectrometry: On the way towards the ideal atomizer. Spectrochim Acta Part B 62(9):846–872. 10.1016/j.sab.2007.05.002

[23] Dědina J (2024) Spectrochim. Acta Part B At Spectrosc 221:107058

[24] Dvořák P, Talába M, Kratzer J, Dědina J (2019) Chem Sci 10:3643 10.1039/c8sc05655b PMC6432262 30996959

[25] Dvořák P, Talába M, Obrusník A, Kratzer J, Dědina J (2017) Plasma Sources Sci Technol 26:085002

[26] Slota A, Svoboda M, Suchopár V, Kratzer J (2023) At. Spectroscopy. 44:207

[27] Vlčková N, Baranová B, Svoboda M, Kratzer J (2024) Spectrochim Acta, Part B 212:106853

[28] Voráč J, Dvořák P, Procházka V, Ehlbeck J, Reuter S (2013) Plasma Sources Sci Technol 22:025016

[29] Dvořák P, Mrkvičková M, Kratzer J (2024) Frontiers in Physics. 12:1408078

[30] Miles RB, Lempert WR, Forkey JN (2001) Laser rayleigh scattering. Meas Sci Technol 12(5):33

[31] Voráč J, Dvořák P, Mrkvičková M (2017) Laser induced fluorescence of hydroxyl (OH) radical in cold atmospheric discharges. In: Britun N, Nikiforov A (eds) Photon counting - fundamentals and applications. IntechOpen

[32] Mrkvičková M, Dvořák P, Svoboda M, Kratzer J, Voráč J, Dědina J (2022) Combust Flame 241:112100

[33] Kramida A, Ralchenko Y, Reader J, NIST ASD Team NIST Atomic Spectra Database (ver. 5.11) (2024) [Online]. Available: https://physics.nist.gov/asd [2024, July 31]. National Institute of Standards and Technology, Gaithersburg, MD

[34] Obrusník A, Dědina J, Dvořák P (2020) J Anal At Spectrom 35:1464

[35] Payling R, Larkins P (2000) Optical Emission Lines Ofthe Elements. Wiley, New York

